# Filamentous Aggregates Are Fragmented by the Proteasome Holoenzyme

**DOI:** 10.1016/j.celrep.2019.01.096

**Published:** 2019-02-19

**Authors:** Rachel Cliffe, Jason C. Sang, Franziska Kundel, Daniel Finley, David Klenerman, Yu Ye

**Affiliations:** 1 Department of Chemistry, University of Cambridge, Lensfield Road, Cambridge CB2 1EW, UK; 2 Department of Cell Biology, Harvard Medical School, Longwood Avenue, Boston, MA 02115, USA; 3 UK Dementia Research Institute at University of Cambridge, Cambridge CB2 0XY, UK

**Keywords:** protein aggregation, tau, proteasome, alpha-synuclein, disaggregation, total-internal reflection fluorescence microscopy

## Abstract

Filamentous aggregates (fibrils) are regarded as the final stage in the assembly of amyloidogenic proteins and are formed in many neurodegenerative diseases. Accumulation of aggregates occurs as a result of an imbalance between their formation and removal. Here we use single-aggregate imaging to show that large fibrils assembled from full-length tau are substrates of the 26S proteasome holoenzyme, which fragments them into small aggregates. Interestingly, although degradation of monomeric tau is not inhibited by adenosine 5’-(3-thiotriphosphate) (ATPγS), fibril fragmentation is predominantly dependent on the ATPase activity of the proteasome. The proteasome holoenzyme also targets fibrils assembled from α-synuclein, suggesting that its fibril-fragmenting function may be a general mechanism. The fragmented species produced by the proteasome shows significant toxicity to human cell lines compared with intact fibrils. Together, our results indicate that the proteasome holoenzyme possesses a fragmentation function that disassembles large fibrils into smaller and more cytotoxic species.

## Highlights

• The proteasome fragments tau and α-synuclein fibrils into small aggregates
• Single-aggregate imaging was used to quantify changes in fibril and aggregate size
• Fibril fragmentation depends on proteasomal ATPase but not proteolytic activity
• Fragmented aggregate species induce cell death more potently than fibrils

## Graphical Abstract

**Figure:**
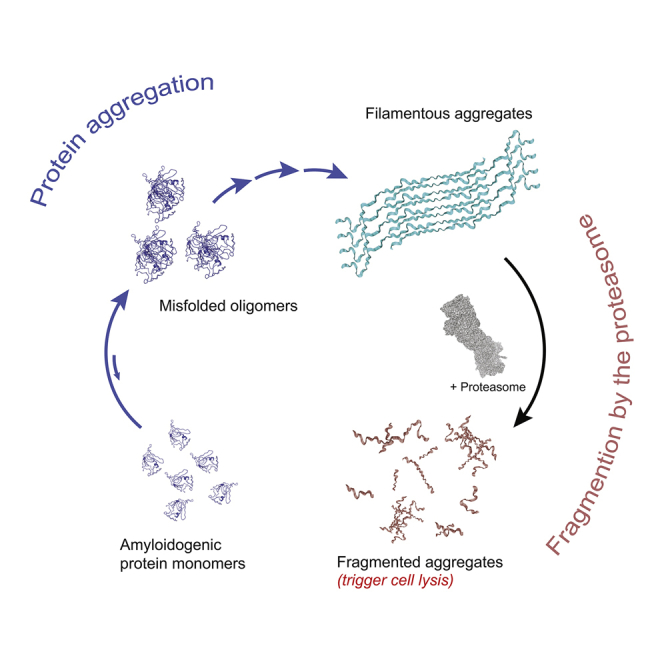

## Teaser

Cliffe et al. show that the proteasome holoenzyme can fragment fibrils assembled from tau and α-synuclein, both of which are associated with neurodegenerative disease. The fragmented aggregate species are structurally distinct from fibrils and more toxic than fibrils when added to cultured cells.

## Introduction

Protein aggregation is often associated with neurodegenerative disorders and aging-related dementia (Goedert, 2015). Alzheimer’s and Parkinson’s diseases are common dementia-like disorders involving aggregation of the distinct amyloidogenic proteins tau and α-synuclein (αS), respectively (Iqbal et al., 2005, Lashuel et al., 2013). The protein tau is suggested to participate in the assembly and stability of microtubules but has also been associated with other functions (Wang and Mandelkow, 2016). αS is able to interact with phospholipids and vesicles and believed to be involved in cellular vesicle trafficking and neurotransmitter release (Bendor et al., 2013). Both tau and αS are largely intrinsically disordered when not associated with other proteins (Schwalbe et al., 2014, Theillet et al., 2016).

Amyloidogenic proteins have a propensity to misfold and oligomerize (Soto, 2003). As oligomers grow in size by addition of protein monomers, conformational changes associated with increased stability take place, eventually resulting in a highly ordered and filamentous arrangement of aggregates that are no longer soluble in the physiological environment (Spillantini and Goedert, 2013). How these aggregates relate to toxicity leading to cell death and ultimately cause pathological disorders remains disputed, although distinct types of aggregates have been found to impede cellular signaling and compromise the integrity of neuronal functions (Goedert and Spillantini, 2017, Haass and Selkoe, 2007, Labbadia and Morimoto, 2015, Selkoe, 2004). The size and type of aggregates may also determine how they are processed by cells and either targeted for degradation or sequestered at distinct cellular sites (e.g., Woerner et al., 2016).

Although the formation of aggregates has been researched extensively, little is known about their removal. Aggregate degradation via both the proteasomal and the lysosomal systems has been described in the literature (e.g., Wang and Mandelkow, 2012, Webb et al., 2003). Although larger aggregates are believed to be cleared by lysosomes, removal of smaller oligomers has been attributed to the proteasome (Rubinsztein, 2006). The 26S proteasome holoenzyme is an abundant multisubunit protein complex responsible for the regulation of many key signaling pathways and general cell homeostasis (Schmidt and Finley, 2014). This complex consists of a cylindrically shaped 20S core particle (CP) and one or two 19S regulatory particles (RPs) that cap the CP at either end (Tomko and Hochstrasser, 2013; modeled in Figure S1A). Degradation activity is provided by several proteases within the interior of the CP, whereas the RP is responsible for the recognition of ubiquitin (Ub)-modified substrates, which are subsequently unfolded and translocated into the CP (Bhattacharyya et al., 2014). Six ATPases arranged in a hexameric ring are found within the base of the RP, which couples ATP hydrolysis to substrate unfolding and translocation through its channel pore. In cells, both tau and αS have been reported to be degraded by the proteasome (Lee et al., 2010, Rott et al., 2011), whereas aggregates assembled from these proteins have not been found to be targeted by the proteasome *in vitro* (e.g., Iljina et al., 2016, Myeku et al., 2016). It is further possible that distinct aggregate conformations of sufficient size and stability may be recognized but not processed by the proteasome and, thus, inhibit its activity, as suggested for tau, αS, amyloid-β, and prion protein aggregates (Kristiansen et al., 2007, Myeku et al., 2016, Tseng et al., 2008, Zhang et al., 2008).

Here we use single-aggregate total internal reflection fluorescence (TIRF) microscopy to show a previously unidentified aggregate fragmentation function of the proteasome holoenzyme that targets fibrils in an Ub-independent manner. Fibrils assembled from full-length tau were predominantly fragmented by the proteasome in an ATP-dependent manner, whereas inhibiting the proteolytic activity of the proteasome had a negligible effect on the fragmentation function. Fragmentation was further confirmed by transmission electron microscopy (TEM), revealing a species that resembled amorphous aggregates following proteasome treatment. This aggregate species was more toxic to cultured mammalian cells than fibrils, triggering a significant level of cell death. Our findings were further confirmed using αS fibrils, suggesting that this fragmenting function is not restricted to targeting tau fibrils. Together, our findings demonstrate the ability of the proteasome holoenzyme to disassemble fibrils, and its activity may be regulated by altering the physiological ratio of the holoenzyme to the free proteasomal core and regulatory particles.

## Results

### The Proteasome Holoenzyme Degrades Monomeric Tau

To study how filamentous aggregates (fibrils) may be processed by mammalian proteasomes (Figure S1A), we purified the holoenzyme or the RP separately from established HEK293T cells (Wang and Huang, 2008; STAR Methods). The purity and integrity of the proteasomes were confirmed using SDS-PAGE and TEM (Figures S1B and S1C). Untagged recombinant full-length tau (isoform 0N4R, modeled in Figure S2A) containing a single Pro274Ser substitution was purified to apparent homogeneity (Figure S2B) and subjected to proteasomal degradation. The Pro274Ser substitution enhances tau aggregation and is commonly used in tauopathy models (Allen et al., 2002). Monomeric tau was degraded by the holoenzyme, as demonstrated by the loss of substrate band intensity over time (Figure S2C). Degradation by the holoenzyme was efficiently inhibited by 50 μM Velcade alone or an inhibitor cocktail (50 μM each of Velcade, MG132, and carfilzomib, all of which target proteasomal proteases) but not by replacement of ATP with a slowly hydrolysable ATP analog, adenosine 5’-(3-thiotriphosphate) (ATPγS) (Figure S2C). This result suggests that the degradation of monomeric tau is dependent on the proteolytic but not the ATPase-catalyzed unfolding-translocation activity of the proteasome and that Velcade is sufficient to fully inhibit proteasomal degradation of tau proteins.

### Tau Fibrils Are Fragmented in the Presence of the Proteasome Holoenzyme

**Figure 1:**
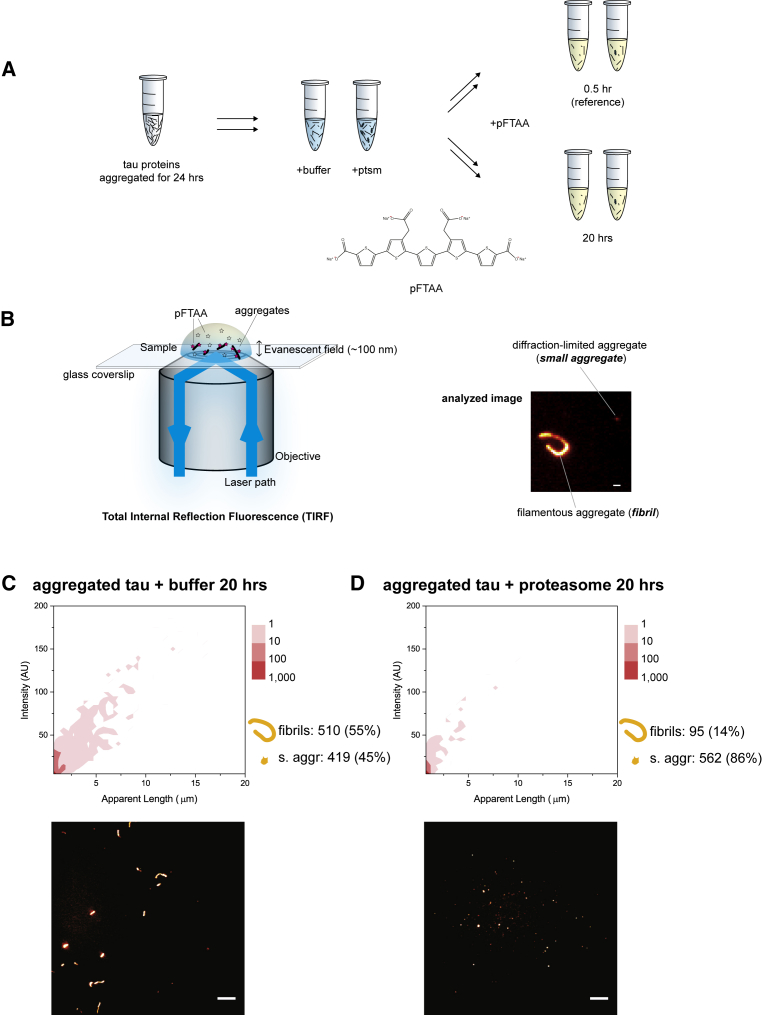
Imaging Fibrils with a Fluorescence TIRF Microscope (A) Recombinant full-length tau was aggregated for 24 h, and aliquots were taken and mixed with the proteasome in an ATP-containing proteasome buffer or with the buffer only as a control. After 0.5 h (starting reference) and 20 h of incubation, each reaction was diluted in an imaging buffer containing pFTAA. The chemical structure of pFTAA, which binds amyloid structures, is shown. (B) Samples were placed on a glass coverslip, excited with a 488 nm laser, and imaged on a custom-built TIRF microscope (see also Figure S3). A typical fibril (length, >1 μm) and diffraction-limited small aggregate (length, <1 μm) are shown. The scale bar represents 1 μm. (C and D) A large amount of fibrils remained present after incubation with the buffer alone (C), whereas treatment with the proteasome holoenzyme resulted in loss of fibrils and an increase in small aggregate count (D; depicted next to the 2D plot). The length of aggregates is plotted against the fluorescence intensity of pFTAA; the frequency is color-coded in the 2D plots. A processed image from each reaction is shown below the respective plots. The scale bars represent 10 μm. Results of three biological repeats (n = 3) performed independently using different protein preparations of tau and proteasome were combined into each plot. The SD between repeats was less than 20% in our TIRF experiments.

We next tested whether aggregates assembled from tau may also be targeted by the proteasome holoenzyme. Fibrils assembled from tau could be reproducibly obtained at similar levels after 24 h of aggregation reaction following established protocols (e.g., Kundel et al., 2018). Aggregated tau samples were treated with the proteasome or an ATP-containing buffer control and subsequently mixed with a second solution containing pentameric formylthiophene acetic acid (pFTAA; Figure 1A), a fluorophore that emits fluorescence upon binding to amyloid structures in aggregates (Brelstaff et al., 2015). We further established an approach to detect aggregated proteins directly on a glass coverslip surface (Figure 1B, left). Our approach does not require prior labeling of tau proteins and permits fluorophores in solution to reversibly bind the aggregates, prolonging imaging lifetime. Aggregates were imaged on a custom-built fluorescence TIRF microscope (Figure S3A) and analyzed using custom-written scripts we developed to assess individual aggregate size and fluorescence intensity, which, in turn, reflects the level of amyloid structures present (Figure S3B; STAR Methods).

The size (apparent length) of individual pFTAA-positive aggregates as detected by TIRF was plotted against their fluorescence intensity and presented in 2D graphs. The level of amyloid structures defined in each aggregate increases proportionally with aggregate size (Figure S3C). Based on the contours of aggregates, we will refer to the large aggregates (length, >1 μm) with distinct shapes and high amyloid structure content as fibrils and those with indistinct morphology because of the resolution (length, <1 μm) as small aggregates (Figure 1B, right; Figure S3D).

Large fibrils (up to 15 μm in length) assembled from tau proteins could still be detected even after 20 h of incubation in degradation buffer without the proteasome. These fibrils constituted about 55% of all aggregates (510 fibril and 419 small aggregate counts; Figure 1C). In comparison, incubation with the proteasome holoenzyme quantitatively removed tau fibrils (95 counts, 7% of the total), whereas the level of small aggregates increased (562 counts; Figure 1D). The standard deviation (SD) from the mean of the experiments was less than 20% for both fibrils and small aggregates. Proteasomes alone did not bind pFTAA and, therefore, could not contribute to any fluorescence signals detected (Figure S4A). An ATP regeneration system was added to both the control and proteasome-treated samples to maintain the ATP concentration during the assay (STAR Methods).

### Fibrils Are Targeted by ATP-Dependent Proteasomal Activity

**Figure 2:**
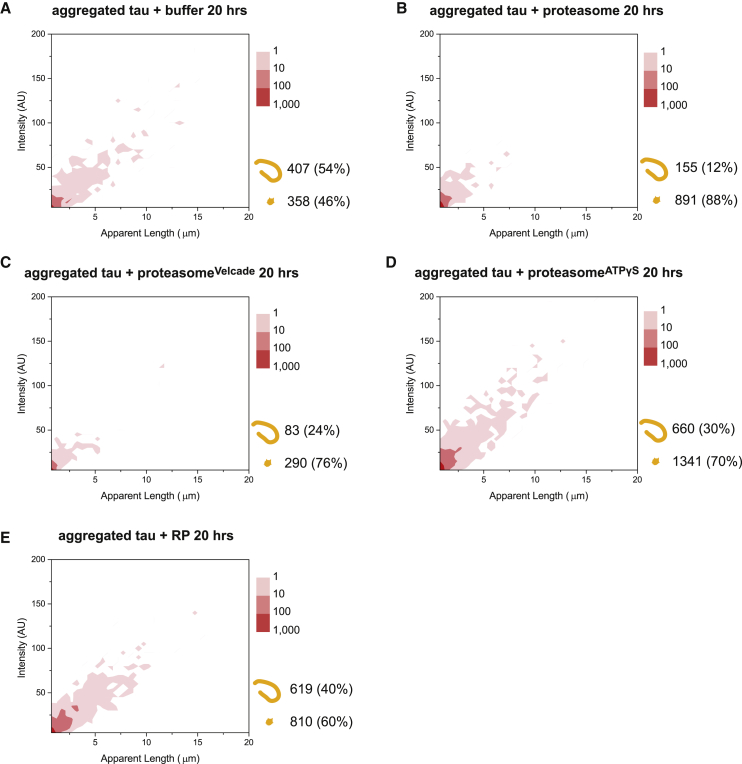
Fragmentation of Fibrils in the Absence of Soluble Tau Proteins (A and B) Aggregated tau samples were centrifuged. The pellet was resuspended in fresh proteasome buffer followed by incubation with (A) buffer control or (B) the proteasome holoenzyme for 20 h and subsequently imaged and presented as described in Figure 1. (C and D) Proteasome holoenzymes pre-treated with (C) Velcade (proteasome^Velcade^) or (D) ATPγS (proteasome^ATPγS^) were subsequently incubated with aggregated tau as in (B). (E) Instead of the holoenzyme, fibrils were also incubated with regulatory particles (RPs) and analyzed as above. Combined results of three independent experiments (n = 3) are shown.

An equilibrium may potentially exist between fibrils, soluble oligomers, and monomers, the last of which could be degraded by the proteasome and, thus, lead to an equilibrium shift favoring fibril disassembly. To validate that fibrils were being targeted by the proteasome, we repeated the experiments in Figures 1C and 1D after soluble tau monomers and oligomers were separated from aggregated proteins by centrifugation (STAR Methods). After removing the supernatant, the pellet containing the fibrils was resuspended with fresh buffer. Following incubation in the buffer control for 20 h, fibrils up to 15 μm in length (407 counts or 54% of the total; Figure 2A) were still detected at a similar level as in Figure 1C. As expected, incubation with the holoenzyme led to fibril fragmentation, resulting in a drop in the level of fibrils (155 counts, 12% of total) and an increase in the number of small aggregates (891 counts, Figure 2b). These results indicate the presence of a proteasomal function that fragments tau fibrils. Plausibly, the proteasome may fragment a single fibril into many small aggregates, at least some of which may be detected by TIRF imaging. The increase in the level of small aggregates here is in agreement with Figure 1 and suggests that fibrils may have been fragmented into smaller species of no more than 1 μm in size. We further addressed the possibility of contamination by canonical chaperones that may be responsible for fibril fragmentation; the fragmenting activity was unaffected by inhibitors of heat shock protein (HSP) 70, HSP90, or VCP, also known as p97 (Figures S4B–S4E). Therefore, the loss of fibrils and the increase in small aggregates appear to be results of proteasomal action.

To gain insights into the proteasomal mechanisms responsible for this fibril-fragmenting function, we repeated the same assay using holoenzymes pre-treated with either Velcade or ATPγS. Fragmentation was largely observed (83 counts of remaining fibrils), even after inhibition of proteolytic activity (proteasome^Velcade^; Figure 2D), further excluding the scenario that proteolytic degradation of tau proteins mediates the loss of fibrils. The number of small aggregates (290 counts) remained at a similar level as the control in Figure 2A. In contrast, compromising the ATP-dependent activity (proteasome^ATPγS^) impeded the fragmenting function, leaving the fibril level largely unchanged (660 counts; Figure 2E). The lower ratio of fibrils (30%) when incubated with proteasome^ATPγS^ is due to the apparent level of small aggregates detected (1,341 counts). These observations together imply that the fragmentation of fibrils relies predominantly on the ATPase activity of the proteasome.

### Integrity of Proteasome Holoenzyme Required for Fibril Fragmentation

Because fibril fragmentation was mainly dependent on proteasomal ATPase activity, we attempted to independently verify our observations using purified free RPs. Unexpectedly, no significant fibril fragmentation was detected in the presence of RP (Figure 2E), and the level of fibrils (619 counts or 40% of the total) remained largely similar to Figure 2A. This result suggests that the integrity of the proteasome holoenzyme may be required to couple functions that are required for efficient fibril fragmentation.

### Distinct Activities of Proteasome Holoenzyme Prevent Tau Aggregation

**Figure 3:**
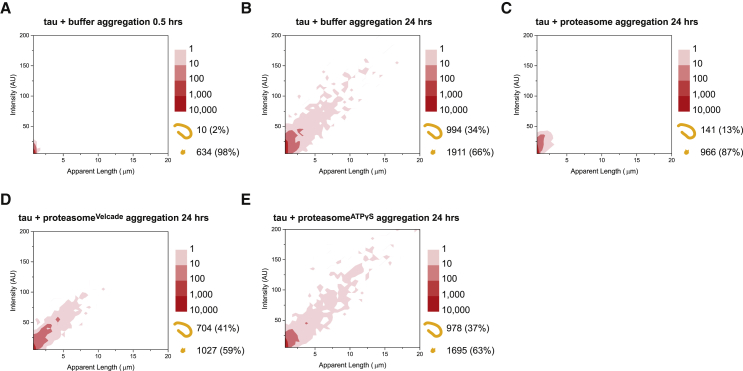
Aggregation of Tau in the Presence of Proteasome Holoenzymes (A and B) Monomeric tau proteins at 2 μM final concentration were mixed with proteasome buffer and imaged after (A) 0.5 h or (B) 24 h, showing a substantial increase in both fibril and small aggregate levels. (C–E) Aggregation of tau in the presence of 40 nM final concentration of (C) untreated, (D) Velcade-treated, and (E) ATPγS-treated proteasome holoenzyme, measured after 24 h of incubation. Each plot contains the cumulative data from three independent measurements (n = 3).

Although the fibril-fragmenting function of proteasome holoenzymes may be relevant to target aggregates that are already assembled (e.g., in a scenario when aggregates enter the host cell from the extracellular environment; Ait-Bouziad et al., 2017, Evans et al., 2018, Takahashi et al., 2015), the proteasomal mechanisms that are involved in clearing cytosolic misfolded proteins to prevent intracellular aggregate formation may be distinct from its fibril-fragmenting function. To further validate the observations in Figure 2 and mimic a physiological scenario where proteasomes are already present during the aggregation process, we attempted to assemble tau aggregates in the presence the holoenzyme. Without the proteasome, fibrils emerged 0.5 h after aggregation start (Figure 3A), and a significant number of fibrils (994 counts or 34% of the total; Figure 3B) was observed after 24 h of reaction. In comparison, aggregation in the presence of holoenzymes restricted the ratio of fibrils (13%, 141 counts) assembled after 24 h (Figure 3C). Selectively inhibiting the proteolytic or ATPase activities of the proteasome partly restored the fibril level (704 and 978 counts, respectively; Figures 3D and 3E), suggesting that both activities are involved in impeding fibril formation from protein monomers. It therefore appears that the proteasomal mechanisms observed here, which are involved in reducing tau aggregation, are distinct from the fibril-fragmenting function in Figure 2, where the proteasomes were introduced to pre-assembled fibrils. Interestingly, proteasome^ATPγS^ incubation enabled both more and large fibrils (over 10 μm) to form compared with proteasome^Velcade^, consistent with a central role of ATPase activity in fibril disassembly.

### TEM Detects Amorphous Structures following Proteasome Treatment

**Figure 4:**
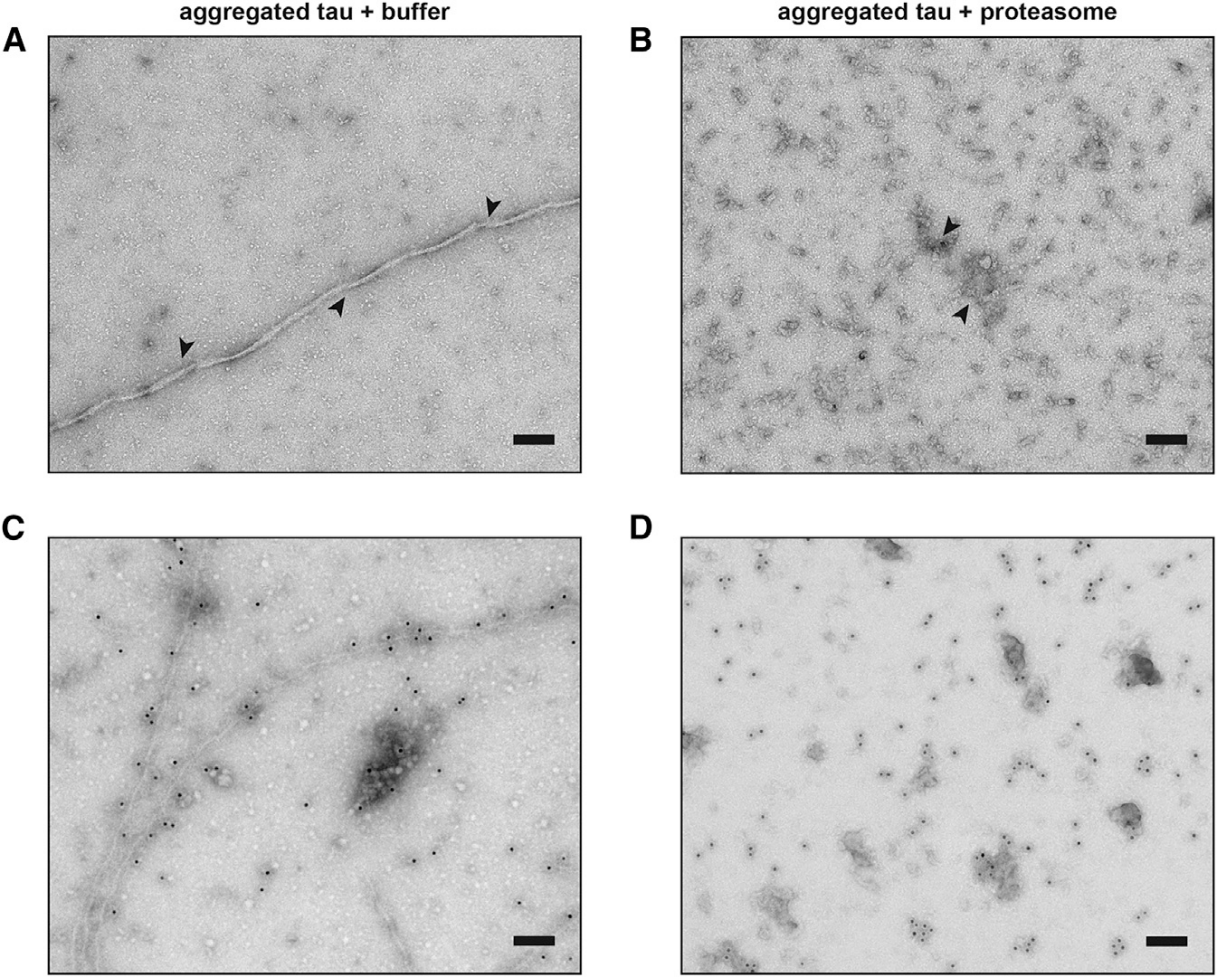
Disordered Aggregates of Amorphous Structures Detected by TEM after Fragmentation by the Proteasome (A and B) Aggregated tau samples prepared as in Figure 2 were incubated with (A) an ATP-containing buffer or (B) proteasome holoenzyme for 20 h, stained with uranyl acetate, and imaged by TEM. Arrows indicate typical aggregated structures. (C and D) Aggregated samples incubated with (C) the buffer control or (D) the proteasome were immunolabeled with an anti-tau antibody, stained with uranyl acetate, and imaged by TEM. The scale bars represent 100 nm. Representative images are shown of at least three independent repeats.

The fragmentation function of the proteasome in Figure 2 was further independently validated using TEM. Fibrils in the absence of the proteasome remained intact after incubation with a buffer control (Figure 4A). These fibrils were lost following proteasome treatment, and only unstructured clusters of proteins resembling amorphous aggregates were detected (Figure 4B), which were not present without proteasome treatment. Because of the intrinsic properties of uranyl acetate, which stains aggregates as well as proteasomes, we repeated these experiments using immunogold labeling with an anti-tau antibody to confirm the presence of tau proteins within these amorphous structures. This approach selectively labeled fibrils in the control sample (Figure 4C) as well as the fragmented species of amorphous structures after proteasome treatment (Figure 4D). These data indicate that tau-containing amorphous structures are formed following fibril fragmentation by the proteasome. Because the dimensions of these amorphous aggregates are mostly less than 1 μm, they are likely to have contributed to the small aggregates observed in Figure 2.

Intact proteasome holoenzymes could be detected under TEM after overnight incubation with the sample (Figure 4B). This suggested that aggregated tau proteins did not affect the integrity of the holoenzyme. Consistently, we detected no quantitative inhibition of the proteasomal peptidase activity against a model substrate, suc-Leu-Leu-Val-Tyr-7-amino-4-methylcoumarin (LLVY-AMC), in the presence of fibrils or by a heparin-containing aggregation buffer (Figure S5A), and incubating fibrils with the active proteasome holoenzyme for 20 h did not affect its ability to hydrolyze ATP (Figure S5B). Although it may be possible that other proteasomal functions, such as Ub-dependent degradation, may be impaired as a result of prolonged interaction with aggregated tau proteins, any such inhibitory effect would not affect interpretation of our fragmentation data.

### Fibril Fragmentation May Increase Cell Lysis

**Figure 5:**
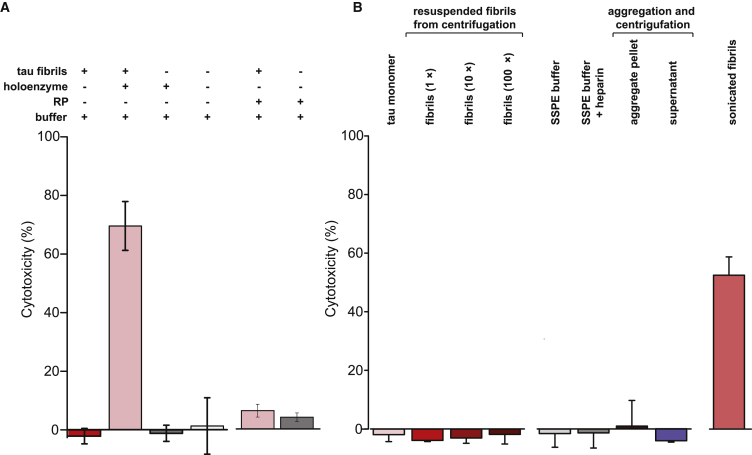
Fragmented Species Trigger Cell Lysis (A) A lactate dehydrogenase (LDH) assay was used to detect lysis of HEK293 cells after incubation with either intact aggregated tau (column 1), the fragmented species (column 2), the buffer alone (column 3), or proteasome holoenzyme alone (column 4). Cells were tested for levels of lysis after incubation with fibrils treated with free RP (column 5) or with free RP alone as a control (column 6). (B) Aggregation buffers and tau fibrils are not toxic to cells. Monomers (column 1) or fibrils (column 2–4) at 1-, 10-, and 100-fold (10× and 100×) of the incubation concentration in (A) were incubated with the cells. Several controls including sodium chloride-sodium phosphate-EDTA (SSPE) buffer alone (column 5), SSPE buffer containing heparin (column 6), the pellet (containing tau fibrils, column 7), or the supernatant fraction (containing soluble aggregates and monomers, column 8) after centrifugation (STAR Methods) were also tested for cell lysis. As a positive control (column 9), fibrils were sonicated briefly and centrifuged to separate insoluble fibrils so that the supernatant could be added to cells at the same calculated concentration. Error bars represent SD of measurements from the mean of three independent experiments (n = 3).

Aggregates assembled from amyloidogenic proteins have been suggested to trigger cell stress and cytotoxicity; for example, by disrupting lipid bilayers (Flagmeier et al., 2017). We hypothesized that the fibril-fragmenting function of the proteasome holoenzyme that resulted in a higher number of small aggregates would also lead to increased cytotoxicity, manifested through cell death (referred to as “cytotoxicity” hereafter). To assess the cytotoxicity associated with fibril fragmentation, we incubated mammalian cells with untreated fibril samples or fragmented species and measured cell viability after 24 h. Viability was monitored using an established assay based on the release of cytosolic lactate dehydrogenase (LDH) into the extracellular medium upon lysis. This assay therefore reports on the toxicity of the aggregate species added to the extracellular environment. No lysis was detected after incubation with fibrils, indicating that the viability of the cells was not affected (Figure 5A, column 1 from the left). In contrast, cells incubated with the fragmented species showed a substantially higher level of cell lysis (Figure 5A, column 2). The reduced cell viability was not caused by the buffer or the proteasome alone, neither of which affected cell lysis (Figure 5A, columns 3 and 4). In comparison, fibrils treated with free RP alone did not show a significant level of cell lysis either (Figure 5A, columns 5 and 6), in agreement with our *in vitro* data in Figure 2E.

Because the total number of aggregates increases after proteasome treatment, we addressed whether a higher concentration of fibrils would also lead to increased cell lysis. Cells incubated with fibrils alone at 1-, 10-, or 100-fold higher concentration than in Figure 5A did not cause any detectable cell lysis compared with the buffer or the tau monomer control (Figure 5B, columns 1–6). We further questioned whether any cytotoxic species might have escaped our detection during the centrifugation procedure, which separated insoluble fibrils from monomers and soluble aggregates. Neither the supernatant nor the pellet samples separated by centrifugation resulted in cell lysis (Figure 5B, columns 7 and 8). A positive control containing fibrils fragmented by sonication resulted in a high level of cell lysis (Figure 5B, column 9), consistent with Figure 5A. Together, these results indicate that the fibril-fragmenting function of the proteasome may have a negative effect on cell viability.

### Conserved Disassembly Action of αS Fibrils by the 26S Proteasome

**Figure 6:**
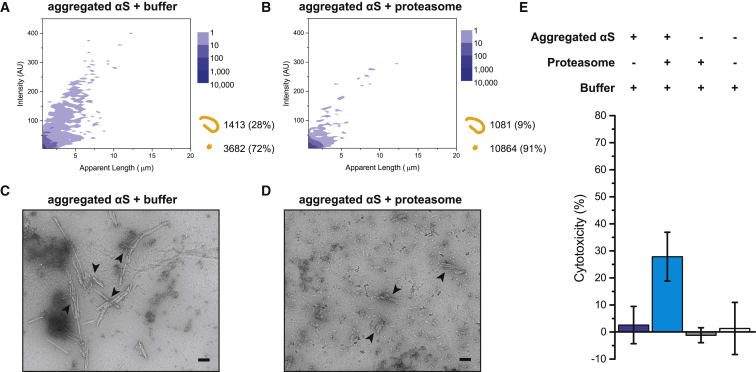
Proteasome Fragments αS Fibrils into Cytotoxic Species (A and B) Aggregates were assembled from αS monomers for 24 h and subsequently incubated with (A) an ATP-containing buffer or (B) proteasome holoenzyme for 20 h and presented in 2D plots as in Figure 2. Combined data of three independent repeats are shown (n = 3). (C and D) The assay was repeated with untreated (C) or proteasome-treated (D) aggregated αS for TEM imaging. Arrows indicate typical aggregated structures. The scale bars represent 100 nm. (E) HEK293 cells incubated with untreated (column 1) and proteasome-treated (column 2) samples of aggregated αS proteins, the buffer (column 3), or the proteasome alone (column 4) were tested for cytotoxicity using the LDH assay as in Figure 5A. Mean values are shown, with error bars representing SD. All experiments were independently repeated at least three times (n = 3) using fresh αS and proteasome preparations.

To test whether the fibril-fragmenting function may also be promiscuous and target other amyloidogenic proteins, we assembled fibrils from recombinant wild-type αS following previously established protocols (e.g., Cremades et al., 2012). Purified monomeric αS is a substrate of the proteasome holoenzyme, and like tau, its degradation is also dependent on the proteolytic but not the ATPase activity of the holoenzyme (Figure S6). Fibrils assembled from αS could be reproducibly detected (1,413 counts or 28% of the total) under the TIRF microscope after 20 h of incubation with the buffer control (Figure 6A). These fibrils were fragmented by the proteasome holoenzyme, resulting in a decrease in the number of fibrils (1,081 counts or 9%; Figure 6B). The lower ratio of fibrils is due to a substantial increase in the number of small aggregates (from 3,682 to 10,864 counts) following proteasome treatment. When untreated or proteasome-treated fibrils were stained with uranyl acetate and imaged by TEM, we detected smaller aggregate species of distinct structures following fragmentation (Figures 6C and 6D). These species were not amorphous and may account for the higher fluorescence signal detected by TIRF imaging after fragmentation of αS fibrils. Similar to tau, fragmented αS entities were also significantly more toxic to cells (∼28% cell lysis) than intact fibrils (∼3% lysis; Figure 6E). Overall, the conservation of the fragmenting function against both tau and αS fibrils suggests that the proteasome holoenzyme may serve as a promiscuous fibril fragmentase able to indiscriminately target fibril structures in general.

## Discussion

In this study we identified a fibril-fragmenting function of the proteasome holoenzyme that targets fibrils assembled from tau and αS proteins in an ATP-dependent manner and does not require its proteolytic activity. This fibril-targeting function makes the proteasome holoenzyme a structurally and functionally distinct fibril-fragmenting enzyme compared with other aggregate-targeting enzymes in the existing family of disaggregases, such as Hsp104, which reverses aggregation of prion proteins (Shorter and Lindquist, 2004), and several HSP70-containing disaggregase complexes (Gao et al., 2015, Nillegoda et al., 2015), one of which has been reported to target αS aggregates (Gao et al., 2015). We further showed that this fragmenting function of the proteasome on pre-assembled fibrils is distinct to the proteasomal actions involved in preventing aggregation of misfolded proteins, which requires both the ATPase and proteolytic activities.

The proteasome has canonically been described to target oligomers and smaller aggregates in solution, whereas degradation of larger fibrils is performed by the lysosomal system (Rubinsztein, 2006). In this study, we provide evidence that amends this view, showing a previously unidentified ability of proteasome holoenzymes with molecular dimensions of ∼15 × 40 nm (PDB: 5GJR) to fragment fibrils up to ∼20 μm in length independently of Ub modification. The recent molecular structure of tau filaments derived from an Alzheimer’s patient reveals extensive β sheet stacking of tau monomers, suggesting a high degree of stability (Fitzpatrick et al., 2017). Our results therefore imply a remarkable ability of the proteasome to target and fragment stable structures several orders of magnitude larger.

Early *in vitro* studies have suggested an ATP-dependent chaperone-like function of the RP with affinity to denatured protein structures without Ub modification (Braun et al., 1999). Recent *in vivo* studies have further found proteasomal recruitment to aggresomes in HEK cells (Hao et al., 2013, Nanduri et al., 2015) and to poly-Gly-Ala repeat (poly-GA) aggregates in neurons (Guo et al., 2018), hinting at plausible proteasome involvement in targeting aggregates. Our findings in this study provide additional evidence suggesting that the ATP-dependent fibril-fragmenting function may also be driven by the unfolding or chaperone-like mechanism, perhaps when substrates cannot be a proteolytic target susceptible to direct degradation.

The current study has focused on the fibril-fragmenting function of proteasome holoenzymes on unmodified recombinant tau and αS. In physiological settings, extensive posttranslational modifications have been identified on tau that affect its aggregation properties or are associated with pathological consequences (Morris et al., 2015, Thomas et al., 2012). Similar effects of various posttranslational modifications have also been reported for αS (Barrett and Timothy Greenamyre, 2015, Hasegawa et al., 2002, Tofaris et al., 2003). The range of aggregate species found *in vivo* that may be targeted by the fibril-fragmenting function of the proteasome or, alternatively, those that are resistant to or inhibitory of this function remain to be studied. Identification of the proteasome holoenzyme with a fibril-fragmenting activity, in addition to its canonical role as a mediator of the degradation of misfolded proteins, could be an interesting amendment to the repertoire of cellular instruments targeting protein aggregation.

## STAR★Methods

### Key Resources Table

**Table:** 

REAGENT or RESOURCE	SOURCE	IDENTIFIER
**Antibodies**		

Mouse monoclonal anti-tau clone 1E1/A6	Millipore	Cat#05-804; RRID:AB_11211556
Rabbit monoclonal anti-αS clone MJFR1	Abcam	Cat#ab138501; RRID:AB_2537217

**Chemicals, Peptides, and Recombinant Proteins**		

pFTAA	Brelstaff et al., 2015	Kind gift from Michel Goedert
Heparin	Fisher Scientific	Cat#BP2524-50
ATP	Sigma-Aldrich	Cat#A1388-5MG
Velcade	Generon	Cat#HY-10227-10mg
MG132	Generon	Cat#10012628-5 mg-CAY
Carfilzomib	Generon	Cat#HY-10455-10mg
ATPγS	Sigma-Aldrich	Cat#A1388-5MG
VER155008	Sigma-Aldrich	Cat#SML0271-5MG
Geldanamycin	Sigma-Aldrich	Cat#SML1278-1ML
NMS-873	Sigma-Aldrich	Cat#SML1128-5MG

**Critical Commercial Assays**		

Malachite Green assay	Abcam	Cat#ab65622
LLVY-AMC	Enzo Lifesciences	Cat#BML-P802-0005
LDH assay	Thermo Fisher	Cat#88953

**Experimental Models: Cell Lines**		

HEK293T cells stably expressing Rpn11-His6-TEV-biotin		Kind gift from Lan Huang

**Recombinant DNA**		

pRK172 vector expressing recombinant tau(0N4R) with P301S mutation		Kind gift from Michel Goedert
pT7-7 vector expressing recombinant asynWT	Addgene	Cat#36046

**Software and Algorithms**		

MATLAB	MathWorks	Version 2016b
ImageJ	National Institute of Health, USA	Version 1.0

**Other**		

SSPE buffer	Thermo Fisher	Cat#AM9770
PBS buffer	Fisher Scientific	Cat#BP399500

### Contact for Reagent and Resource Sharing

Further information and requests for resources and reagents should be directed to and will be fulfilled by the Lead Contact, Yu Ye (yy308@cam.ac.uk).

### Experimental Model and Subject Details

#### Plasmids and Cell Lines

All plasmids and cell lines used in this study are listed under Key Resources Table. Plasmids expressing untagged full-length tau (isoform 0N4R) containing a single Pro274Ser substitution or wild-type α-synuclein (αS) in pT7-7 vectors were transformed into BL21 cells. BL21 (DE3) pLysS E. *coli* cells were grown at 37°C in LB media containing 100 μg/ml ampicillin under shaking conditions and protein expression was induced at an O.D. of 1.0 using 1 mM IPTG for 4 hr at 20°C.

HEK293T cells (female) stably expressing a fusion construct coding for Rpn11-His_6_-TEV-biotin were used for proteasome purification. The cells were cultured in DMEM (Thermo Fisher Scientific) supplemented with 10% fetal bovine serum (Sigma) and 1% penicillin/streptomycin (Thermo Fisher Scientific). A dedicated incubator at 37°C was used to incubate the cells in an atmosphere of 5% CO_2_ in air. Monolayers of cells at > 90% confluence were collected using a cell scraper for subsequent purification steps.

### Methods Details

#### Protein purification

BL21 cells expressing recombinant proteins were collected by centrifugation at 4000 × g and then resuspended in ice-cold Lysis buffer (50 mM MES pH 6.5, 2.5 mM TCEP, 1 mM AEBSF). Lysis of cells was carried out by sonication and the lysate was cleared by centrifugation at 23000 × g for 30 min at 4°C. The pH of the cleared lysate containing tau was gradually reduced to 4.5 and incubated on ice for 10 min before repeating the centrifugation at 23000 × g to clear the lysate of precipitants. The supernatant containing tau was filtered and subsequently loaded onto a ResourceS ion exchange column (GE Healthcare) and eluted with a linear NaCl gradient. Eluted fractions containing tau were identified by SDS-PAGE and loaded onto Superdex 16/60 (GE Healthcare) gel filtration column for a final purification step (Figure S2B). For αS purification, the cleared lysate was incubated in boiling water for 15 min followed by another centrifugation procedure at 23000 × g for 30 min at 4°C. The αS supernatant was then loaded onto a ResourceQ anion exchange column and eluted with a linear NaCl gradient. Fractions containing αS were loaded onto Superdex 16/60. The eluted fractions from gel filtration were examined by SDS-PAGE and fractions judged pure were concentrated and flash-frozen.

Purification of mammalian proteasome holoenzymes or the free regulatory particle (RP) was carried out as described elsewhere (Wang and Huang, 2008). Briefly, HEK293T (female) cells were resuspended in Proteasome buffer (50mM Tris [pH7.5], 5 mM ATP, 5 mM MgCl_2_) and lysed using a dounce homogenizer. Cell lysate was cleared by centrifugation at 1500 × g for 10 min and the supernatant was incubated with 2 mL NeutrAvidin beads overnight at 4°C. On the next day, beads were washed with TB buffer (50mM Tris [pH7.5], 5 mM ATP, 5 mM MgCl_2_, 10% glycerol) and bound holoenzymes were eluted after 3 hr of incubation with 6 μl TEV protease (Sigma) at 30°C. For RP purification, the beads were first washed with TBN buffer (TB buffer containing 800 mM NaCl) to release the CP from the bound RP and then with additional TB buffer prior to the TEV protease cleavage step. Subsequent purification steps were the same as for the holoenzyme.

#### Aggregation assays

Aggregation reactions for tau were set up at 2 μM final concentration in SSPE buffer (10 mM Na_3_PO_4_ (pH 7.4), 150 mM NaCl, 1 mM EDTA, 0.02% Sodium Azide) in the presence of 2 μM heparin (5000 Da, Fisher Scientific) at 37°C(Kundel et al., 2018). For αS aggregation, protein was diluted to 70 μM final concentration in PBS buffer (Fisher Scientific) containing 0.02% Sodium Azide and performed under shaking conditions at 37°C(Cremades et al., 2012). Both tau and αS were aggregated for 24 hr and used immediately for subsequent reactions. All buffers used in our assays were pre-filtered with 0.02 μm filters.

Aggregation reactions in Figure 3 contained 2 μM and 40 nM final concentration of tau and proteasome holoenzyme, respectively, in the Proteasome buffer with an ATP regeneration system (20 mM Creatine phosphate and 5 μM Creatine kinase final concentration) and initiated with 2 μM heparin at 37°C as described above.

#### Proteasome assays

Aliquots were removed from aggregation reactions after 24 hr and incubated with the proteasome. The final reactions contained 8 μl of 200 nM proteasome, 8 μl of the aggregated tau or αS substrate, 5 μl of 10 × Proteasome buffer, 2.5 μl of a 20 × ATP regeneration system (2 M Creatine phosphate and 100 μM Creatine kinase) and ddH_2_O to make up 50 μl final reaction volume. The reactions were performed at 25°C to avoid further aggregation. After 0.5 hr (used as a reference) and 20 hr of incubation, 1 μl aliquot was removed from each reaction and serially diluted 50-fold in PBS buffer containing 30 nM pFTAA for TIRF imaging (see below). For experiments in Figures 2, 3, 4, 5, and 6, prior to the proteasome treatment step, aggregated samples were centrifuged on a benchtop centrifuge at maximum velocity for 30 min. The supernatant was subsequently removed and resuspended with an equal volume of Proteasome buffer before incubation with the proteasome. In control reactions, the proteasome was replaced with an equal volume of the Proteasome buffer. The distinct catalytic activities of the proteasome were inhibited by pre-incubation with Velcade or ATPγS for 5 min in room temperature before mixing with the substrate. The final concentrations of Velcade and ATPγS used were 50 μM and 9 mM, respectively. No ATP was added to the buffer in reactions containing ATPγS. Proteasomes pre-treated with VER155008, Geldanamycin and NMS-873 were prepared the same way as Velcade and used at 50 μM final concentration.

#### LLVY-AMC and Malachite Green assays

LLVY-AMC (Enzo Lifesciences) was used at 1 μM final concentration and incubated either with 40 nM final concentration of proteasome alone, with the proteasome in presence of 320 nM heparin or with 320 nM aggregated tau proteins. The sample was excited at 340 nm and emission detected over time on a fluorimeter (Cary Eclipse) at 440 nm. ATPase kit containing the Malachite Green assay was purchased from Abcam (ab65622). For experiments in Figure S5B, 40 μL Proteasome assays set up as described above were mixed with 6 μL of the malachite green reagent and incubated for 15 min before measuring the O.D. at 650 nm on a microplate reader. Free phosphates were used to establish a linear standard curve to calculate phosphate concentrations from colorimetric readings.

#### Degradation assays of protein monomers

Degradation assays of protein monomers were set up mixing 8 μl tau or αS at 2 μM or 70 μM, respectively, with 8 μl of 200 nM proteasome and H_2_O to make a final 50 μl reaction volume. At indicated time points, 6 μl of samples were removed and quenched with 6 μl LDS buffer and stored by flash-freezing for subsequent protein gel electrophoresis. Protein samples were separated by 4%–12% Bolt SDS-PAGE gels (Invitrogen) and transferred to PVDF membranes using Trans-blot Turbo (Biorad) semi-dry transfer system as per manufacturer’s protocol. Membranes were incubated with primary antibodies against tau (1E1/A6, Millipore) or αS (MJFR1, Abcam) overnight. Protein bands were detected using secondary anti-mouse or anti-rabbit antibodies tagged with Alexa647 (Invitrogen) and scanned on a Typhoon Imager.

#### TIRF imaging

Prior to imaging, glass coverslips (0.13 mm thickness, VWR International) were cleaned with an argon plasma (PDC-002, Harrick Plasma) for 1 hr. A multi-well chambered coverslip (Sigma-Aldrich, GBL103350-20EA) was adhered to each glass coverslip to allow for imaging of multiple samples. For the imaging of αS samples, each well was coated with 0.01% poly-L-lysine (MW 150,000-300,000, Sigma-Aldrich) for 15 minutes, then washed three times with sterile-filtered PBS buffer. For tau samples, the glass coverslips were untreated. The imaging concentrations for tau and αS were 20 nM and 100 nM, respectively, of the calculated monomer concentration. Samples were imaged in the presence of 30 nM pFTAA dye (kind gift from Michel Goedert) in PBS buffer, using a home-built total internal reflection fluorescence (TIRF) microscope as shown in Figure S3A.

#### TEM imaging

Samples for TEM imaging were prepared as for TIRF imaging and applied onto a carbon-coated 400 mesh copper grid (Agar Scientific). Proteasome samples were applied at 100 nM final concentration. Samples were stained with 2% (w/v) uranyl acetate for 1 min and subsequently washed twice with ddH_2_O. TEM images were acquired using Tecnai G2 microscope (13218, EDAX, AMETEK) operating at an excitation voltage of 200 kV.

#### Cytotoxicity assays

Fibrils treated with either the Proteasome buffer or with the proteasome holoenzyme (described in the Proteasome Assays section) were added in triplicate to confluent HEK293A cells grown in 96-well plates. The cells were subsequently incubated at 37°C for 24 hr in 200 μl final media volume, from which 100 μl was removed for LDH assay (Thermo Fisher). We followed manufacturer’s protocols and added 100 μl of the supplied Reaction Mixture, which contains the substrate for LDH activity detection, to the media. After 30 min, the reactions were quenched with the Stop Buffer (supplied with the kit) and the absorbance at 480 nm was measured on a plate reader. Lysis media supplied by the manufacturer was then added to each well to establish the maximum level of cell lysis. Assays were repeated at least three times using a new protein preparation each time. Both Velcade and ATPγS alone were found to be toxic to cells when added to the cells at the concentration used for proteasome inhibition, as significant levels of cell lysis was observed.

### Quantification and Statistical Analysis

#### Image analysis and statistics

Images were recorded for 100 frames with 50 ms exposure time. For each sample, 9 fields of view were typically acquired and then analyzed. These raw images collected by the TIRF microscope (see Figure S3) were analyzed to count the total number of fibrils and aggregates, their size and fluorescence intensity. The number of fibrils and aggregates counted was then directly compared between different experiments.

Individual image data were averaged over all the frames by the average intensity projection at z axis using ImageJ (National Institute of Health, USA) and then subjected to image processing. A custom-written MATLAB script (MathWorks) was used to analyze the averaged images. Individual images were first top-hat and bpass filtered to remove the camera noise and partitioned into a foreground and a background. To identify particles, the foreground was blurred using a 2D Gaussian filter with a threshold applied based on the original pixel intensity with a criterion of 2% intensity above the background, and then established boundaries for individual particles. The particle length was measured by thinning boundaries of individual particles and thus calculated with an image pixel size of 235 nm for our TIRF setup. To eliminate the background effect in intensity calculation, signal-to-background ratio (SBR) was introduced to correct pixel intensity, where each pixel’s SBR is defined as:

$$SBR=\frac{Intensity\;above\;the\;background}{Background}$$

For a given particle, its corrected intensity is the sum of each pixel’s SBR values within its boundary. We typically detect over 800 fibrils and aggregates for each experiment. Increasing the number of images does not affect the ratio between fibrils and aggregates, indicating that our sample size was sufficiently large to be representative.

#### Statistical analysis

Mean and stand deviation from three biological repeats (n = 3) of each assay are shown in Figure 5 and Figure 6E. For TIRF measurements in Figure 2 and Figure 3, results of three biological repeats (n = 3) performed independently using different protein preparations of tau and proteasome were combined into each plot. The standard deviation between repeats was less than 20%.

## Supplemental Information

Document S1. Figures S1–S6

Document S2. Article plus Supplemental Information
