# Supplementary material for: Filamentous Aggregates Are Fragmented by the Proteasome Holoenzyme
Source: Cell Rep. 2019 Feb 19;26(8):2140–2149.e3. doi: 10.1016/j.celrep.2019.01.096 (PMC6381791; doi:10.1016/j.celrep.2019.01.096)
Supplement: Document S1. Figures S1–S6 [file mmc1.pdf]

Cell Reports, Volume *26*

Supplemental Information

Filamentous Aggregates Are Fragmented by the Proteasome Holoenzyme

Rachel Cliffe, Jason C. Sang, Franziska Kundel, Daniel Finley, David Klenerman, and Yu Ye

Supplementary Figure 1

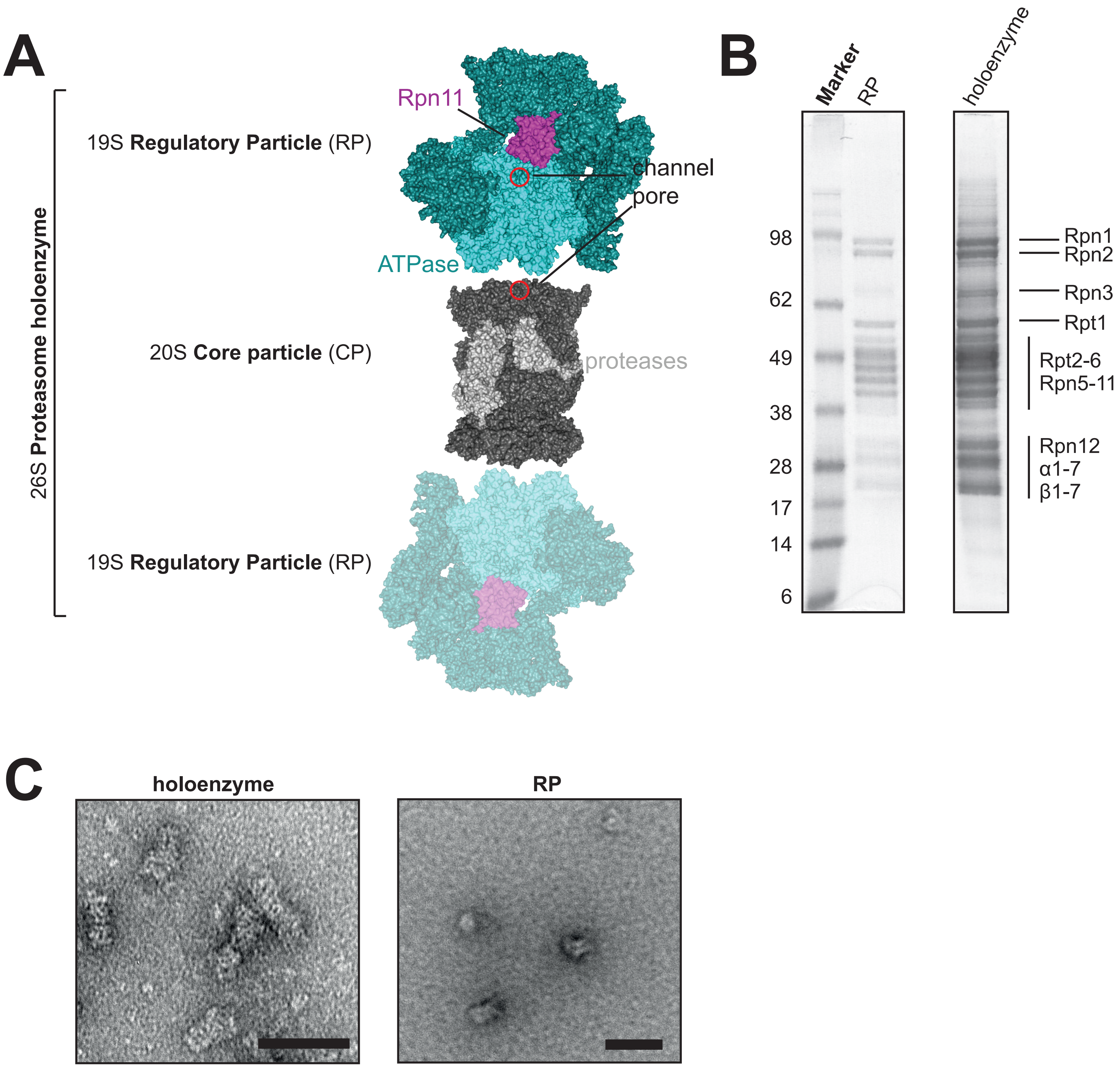

SFigure 1. Purification of mammalian proteasome assemblies, related to **Figures 1 - 6**.

(A) A molecular model of the 26S proteasome holoenzyme (pdb-id: 5GJR) comprising one 20S core particle (CP, grey) capped at both ends with a 19S regulatory particle (RP, cyan). The ATPase subunits (light cyan) of the RP and the proteases of the CP (light grey) are highlighted. Rpn11 (magenta), a subunit of the RP, is modified at the C-terminus with a biotin-tag and used for affinity purification. **(B)** Purified RP (*left*) and proteasome holoenzyme (*right*) were resolved by SDS-PAGE and visualized by Coomassie staining. **(C)** TEM visualization of the holoenzyme (*left*) and the RP (*right*). Scale bars indicate 100 nm.

Supplementary Figure 2

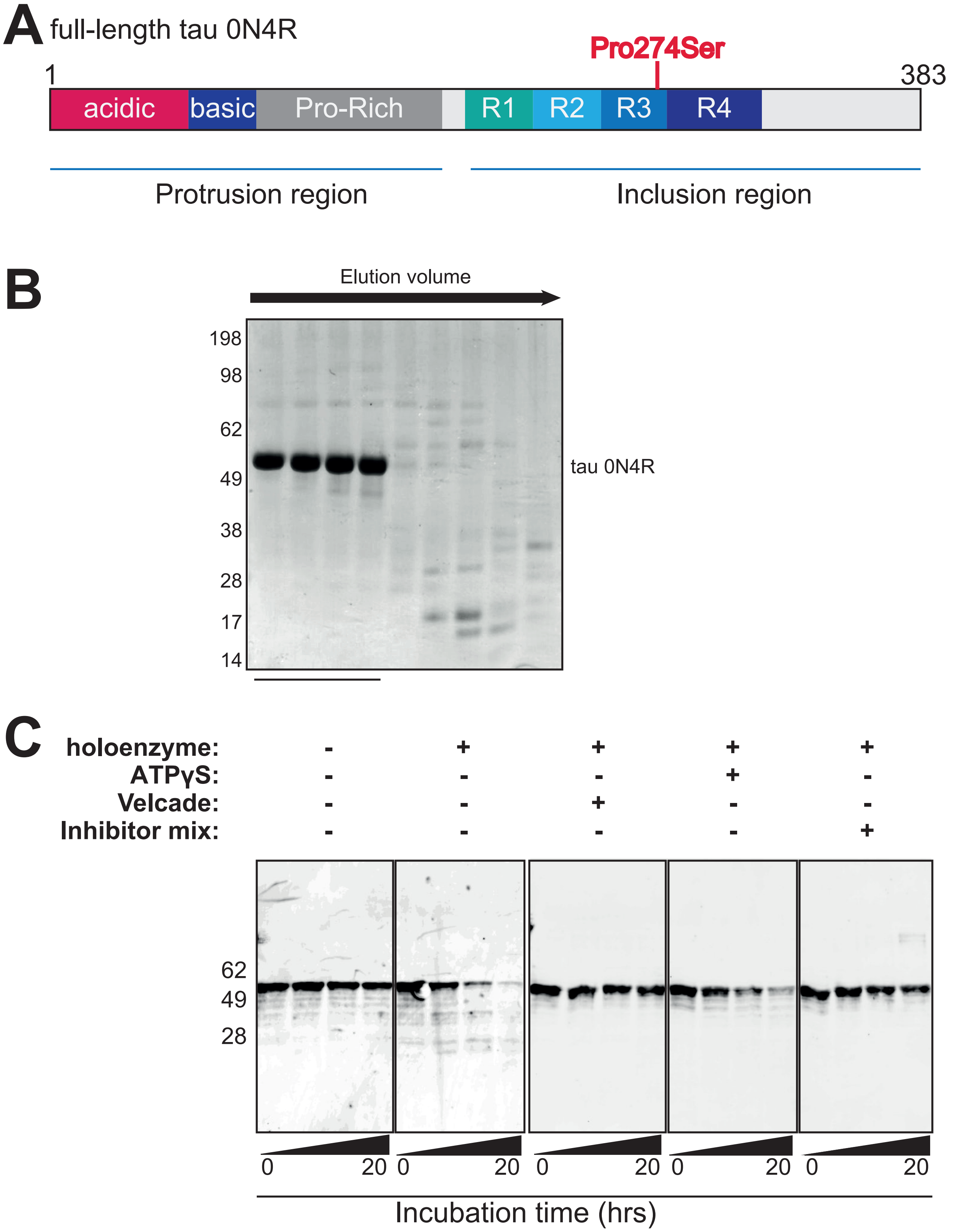

SFigure 2. Recombinant full-length tau monomers are degraded by the proteasome, related to **Figures 2 - 5**.

(A) Model of the primary structure of full-length tau (isoform 0N4R, 383 Aas). The protein consists of an N-terminal protrusion region that does not participate in the assembled fibril structure, with distinct sequences that are acidic, basic and proline-rich. The tetra-repeat sequence within the C-terminal inclusion region is important to the assembly of tau aggregates. The tau proteins used in this study contain a single Pro274Ser substitution that enhances aggregation. **(B)** Pure recombinant tau proteins were obtained after a final gel filtration step. Fractions pooled for subsequent assays are marked. **(C)** From left: monomeric tau was incubated with the Proteasome buffer, the holoenzyme or holoenzyme pre-incubated with 50 µM Velcade, 30 mM ATPγS or an inhibitor cocktail. Aliquots from each reaction were quenched after 0, 5, 10 or 20 hrs, separated by SDS-PAGE and detected by immunoblotting against tau (clone 1E1/A6). The inhibitor cocktail contained 50 µM each of Velcade, MG132 and Carfilzomib.

Supplementary Figure 3

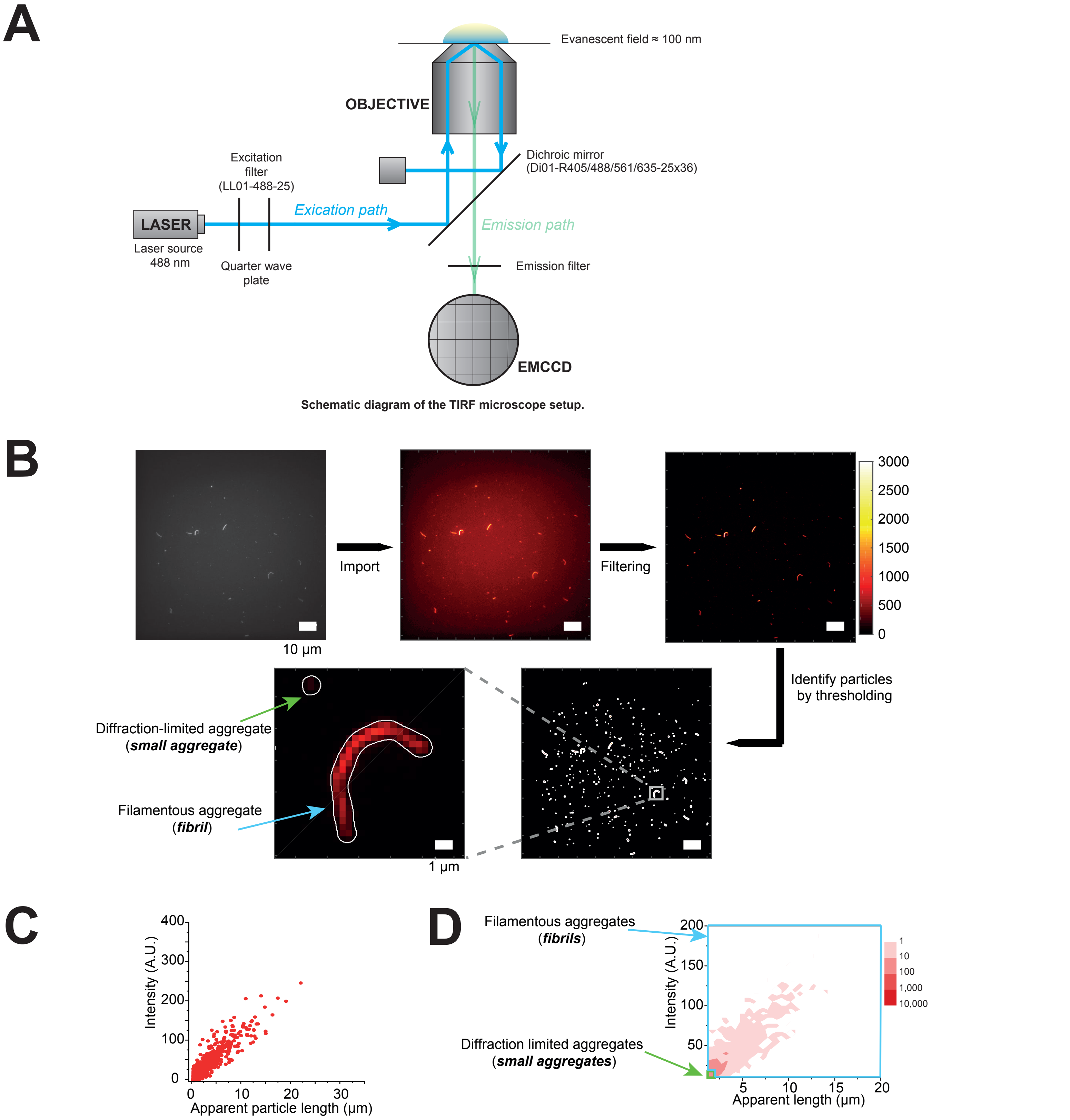

SFigure 3. Imaging aggregated tau proteins using a custom-built TIRF microscope, related to **Figures 2, 3** and **6**.

(A) A model of the TIRF microscope set-up with a 488 nm laser (Cobolt MLD) directed to a 1.49 numerical aperture objective (APON60XO TIRF, Olympus) mounted on an inverted Nikon Eclipse Ti microscope. Fluorescence was collected by the same objective and separated from the returning TIRF beam by an appropriate dichroic (Semrock), and passed through appropriate emission filters (FF03-525/50-25, Semrock). Hardware was controlled using custom-written scripts for MicroManager (NIH) and images were recorded on an EM-CCD camera (Evolve 512Delta, Photometrics) with 235 nm image pixel size. **(B)** A schematic representation of the analysis workflow, where raw images were averaged over 100 frames, the fluorescence signals filtered and individual particles subsequently identified. Each particle is quantified by its size (apparent length as detected by TIRF) and fluorescence intensity (pFTAA binding). We define large filamentous aggregates (length > 1 μm) as ‘fibrils’ and near diffraction-limit aggregates (length < 1 μm) as ‘small aggregates’. Zoom-in shows a typical fibril next to a small aggregate according to the assigned criteria. Sizes of the scale bars are indicated below each row of images. **(C)** Each identified particle is plotted with respect to its length and fluorescence intensity. **(D)** The frequency of particles are binned together (length bin size 1, intensity bin size 5) and color-coded in the 2D plot. Regions are boxed according to where fibrils (cyan) and small aggregates (green) are found.

Supplementary Figure 4

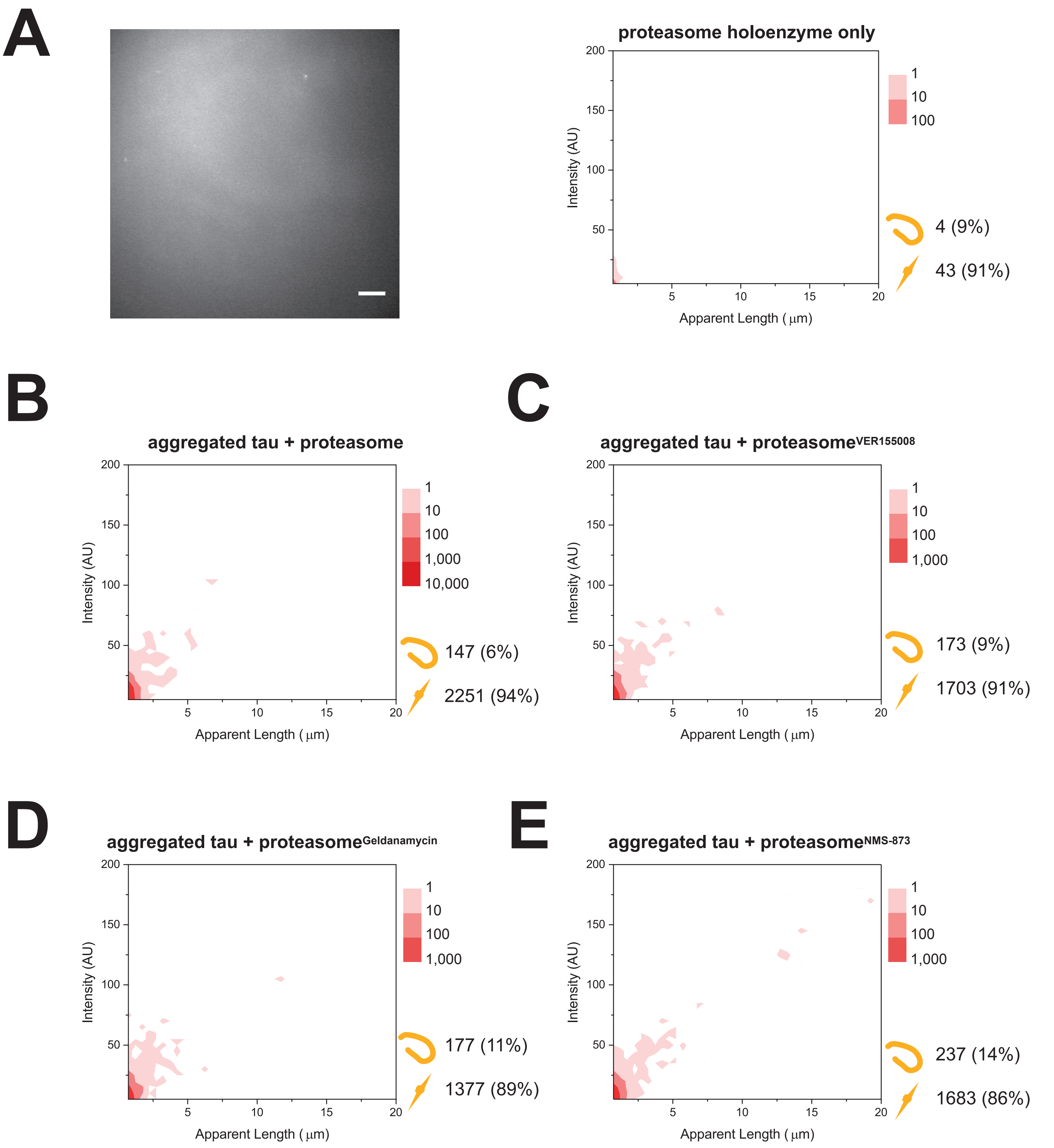

SFigure 4. Fragmentation of fibrils is specifically due to the proteasome, related to **Figure 2**.

(A) The fluorescence level of proteasome holoenzyme alone in the absence of any aggregated sample is very close to the background level (*left*), with an insignificant amount of particles detected (*right*). **(B-E)** The proteasome holoenzyme was pre-treated with **(B)** an ATP-containing buffer control, **(C)** 50 μM Geldanamycin (HSP90 inhibitor), **(D)** 50 μM VER-155008 (HSP70 and HSC70 inhibitor) or **(E)** 50 μM NMS-873 (VCP/p97 inhibitor) and subsequently incubated with aggregated tau samples as in **Figure 2**. Cumulative data of three independent repeats (n = 3) are presented in each plot.

Supplementary Figure 5

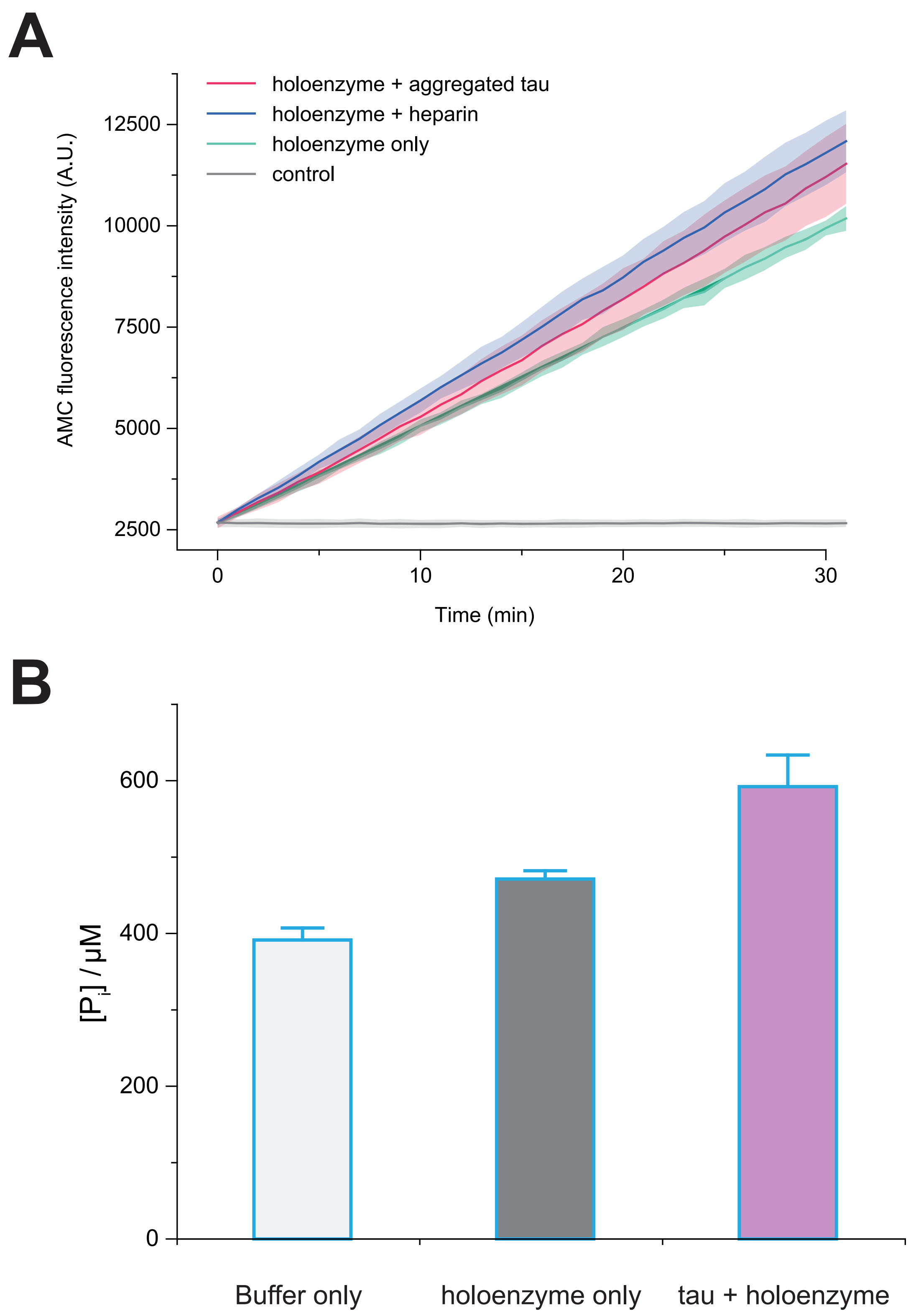

SFigure 5. No quantitative inhibition of the proteolytic and ATPase activities of the proteasome, related to **Figures 2 - 5**.

(A) Proteasome holoenzymes were pre-treated with an ATP-containing buffer control (green), with the buffer containing 10 µM Heparin (blue), or with aggregated tau samples (red) in the same buffer. Proteolytic activity against a model fluorescent substrate LLVY-AMC was subsequently measured from its fluorescence emission (mean and standard deviation, n = 3). As a control, holoenzyme was pre-treated with 50 µM Velcade (grey). **(B)** ATP hydrolysis activity was measured using the malachite assay, which detects the concentration of free phosphates in the buffer. The level of free phosphates detected in the buffer alone (light grey), holoenzyme alone (brown) or holoenzyme with aggregated tau (magenta) incubated for 20 hrs as in **Figure 2** are shown (mean and standard deviation, n =3).

Supplementary Figure 6

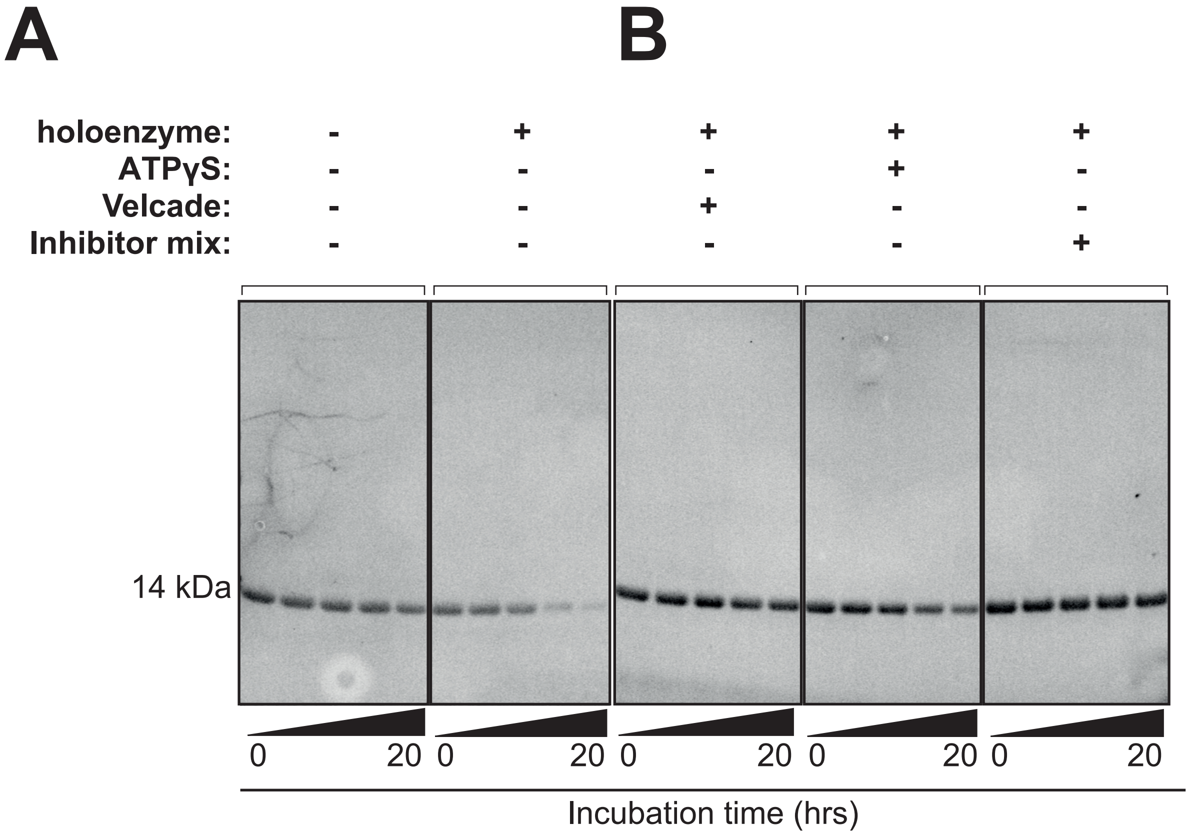

SFigure 6. Recombinant full-length αS monomers are degraded by the proteasome, related to **Figure 6**.

(A) Monomeric αS incubated with buffer control (*left*) or the holoenzyme (*right*). **(B)** From left: Velcade-, ATPγS, or inhibitor cocktail-treated holoenzymes were incubated with monomeric αS. Aliquotsfrom each reaction were taken at 0, 3, 5, 10 or 20 hrs after incubation, quenched and separated by SDS-PAGE. Changes in the αS level were detected by immunoblotting against αS (clone MJFR1). Experiments performed as in **SFigure 2C**. Representative data of at least three independent repeats.
